# Nurses' Eight Hours' Work

**Published:** 1920-01-31

**Authors:** 


*To the Editor of* THE HOSPITAL.

### NURSES' EIGHT HOURS' WORK.

*To the Editor of* THE HOSPITAL.

SIR,—Personally, I think all nurses, past, present, and future, cannot but admire and feel grateful to "Ierne" in her ceaseless effort for the welfare of nurses, and incidentally patients. None of us are capable, when very tired, of doing our best. The long hours of hospital, while curing patients or helping those already ill, has often done much to wreck the constitution of women in their early youth, who started their hospital career very physically fit.

Now I am older and have a bigger outlook on life, I cannot express how anxious I am to see the working days of probationers shorter. I speak the more feelingly as I am unable to continue working, owing to ill-health.

We all know a large hospital did not let you sign an agreement for training till you proved physically fit. Think of the strain on the health of a young adult on night duty. We were on duty, without any break from the wards, for twelve hours; often we worked anxiously the whole night through. Apart from our patients and wards, we had much cleaning to do. Our food was eaten in the wards, sometimes under difficulties, occasionally merely a cup of tea only, owing to pressure of work. At the end of such nights, having with luck got to our bedrooms by 9.30 A.M., we had beds to make, bedroom to sweep and dust, dinner 10 A.M.; twice a week by 10.30 we were due in a class-room to study! Naturally it was only the exception for us not to fall asleep the greater part of the time, and were rudely awakened by the home sister on duty, whose job was to keep us awake, and often report us when it proved futile. Twice a week we were called an hour earlier than usual, once to go to an instruction class and once a lecture. Unless we made the effort to get a cup of tea ourselves we had no meal of any sort till these were over. It sounds to me like the dark ages.

Private nursing wants reforming badly. The houses vary as do patients, as much as East and West. The need for recognised rest and recreation is great.

Maternity work I have said little of, but both in hospital and private maternity nursing more rest for nurses out to earn their living is urgent.

Think of the waste of training women and then getting well-trained, capable women laid aside in very early middle life (thirty-three to forty), because one has used up her vitality so extravagantly it has come to an untimely end. It is so false an economy as to be a disgrace to intelligent heads of the profession.

My renewed thanks and good luck to "Ierne."

Yours faithfully, JUSTICE.

